# Professional Appointments

**Published:** 1858-10

**Authors:** 


					﻿NEW YORK STATE INEBRIATE ASYLUM.
The State of New York enjoys the honor of being the first in
our country to appropriate the means for establishing a State
institution for the reception and treatment of confirmed inebriates.
It has been located in the beautiful 'village of Binghamton, in
the southern part of the State, and the corner stone was laid on
• the 24th of September last. Rev. Dr. Bellows, Dr. John W.
Francis, Hon. Edward Everett and Hon. D. S. Dickinson, took
part in the exercises.

				

